# Intrapancreatic autologous stem cell therapy for type 1 diabetes – an experimental study

**DOI:** 10.1097/MS9.0000000000000837

**Published:** 2023-07-28

**Authors:** Sagar Jawale

**Affiliations:** Jawale Hospital, Jalgaon, Maharashtra, India

**Keywords:** intrapancreatic stem cell therapy for type 1 diabetes, reversal of autoimmunity in type 1 diabetes by stem cell therapy, stem cell therapy, stem cell therapy for type 1 diabetes, type 1 diabetes

## Abstract

**Introduction:**

Type 1 diabetes, also known as juvenile diabetes or insulin-dependent diabetes, is a chronic autoimmune condition in which the pancreas makes little or no insulin leading to resultant hyperglycemia. The incidence of type 1 diabetes in India is 0.26 per 1000 children.

**Materials and methods:**

The author treated 25 patients with type 1 diabetes with autologous intrapancreatic stem cell therapy in the last 5 years. A group of 26 patients of type 1 diabetes with conventional treatment of insulin injections was put as a control group in the same period.

**Results:**

The results of the therapy group were substantially superior to the control group, which came statistically highly significant. The variable compared were weight gain, the daily requirement of insulin and its drop after therapy, the rise of C-peptide levels and drop in anti-glutamic acid decarboxylase antibody, drop in HbA1c levels, and drop in fasting and postprandial blood sugar.

**Discussion:**

When stem cells are given intravenously, the majority are engulfed by the lungs and only a small fraction is delivered to the pancreas. When injected into the pancreas, through its arterial blood supply, due to the larger size and irregular shape of stem cells, they are retained in tissue spaces and do not escape from the venous side, thus achieving far higher concentration in the pancreas compared to the intravenous route.

**Conclusions:**

Intrapancreatic stem cell therapy for type 1 diabetes is safe, affordable, and effective. It has the potential to become a viable treatment option for type 1 diabetes patients.

## Introduction

Highlights

- Intrapancreatic autologous bone marrow-derived stem cell therapy for type 1 diabetes.
- It implants stem cells into the pancreas through the pancreatic artery.
- The therapy group’s results were superior to the control group and were statistically significant.
- Stem cells injected into the pancreas achieve far higher concentrations compared to the intravenous route.
- The therapy was safe and effective in the long term for the treatment of type 1 diabetes.

Type 1 diabetes, also known as juvenile diabetes or insulin-dependent diabetes, is a chronic autoimmune disease that occurs when the immune system attacks and destroys the insulin-producing beta cells in the pancreas. The resulting insulin deficiency causes high blood glucose levels, which, if left untreated, can lead to serious complications such as blindness, kidney failure, and heart disease. Despite advancements in diabetes management, there is still no cure for type 1 diabetes. This paper explores the current research on intrapancreatic autologous stem cell therapy for type 1 diabetes and its potential as a promising alternative to traditional diabetes management. In a study and meta-analysis, data were extracted from 193 articles between 1990 and 2019. It showed that the incidence of type 1 diabetes in continental subgroups (Asia, Africa, Europe, and America) was 15 per 100 000, 8 per 100 000, 15 per 100 000, and 20 per 100, respectively. The international incidence of continental subtypes of type 1 diabetes in the above regions was 6.9 per 10 000, 3.5 per 10 000, and 12.2 per 10 000, respectively^1^. The incidence of type 1 diabetes in India is 0.26 per 1000 children^2^. The disease is treated daily by insulin injection and dietary control of sugar and sugar-containing foods. Islet cell transplantation is an FDA-approved treatment at present for long-term remission. Daily insulin injections and their cost are both unacceptable to most children. Islet cell transplantation is very costly, and the patients remain in remission only for 2–5 years^3^. After 3–5 years, most of the patients are back on insulin injections. After islet cell transplantation, patients have to be put on immunosuppression for a long time. Its side effects and cost are the downside of this particular treatment. There are many clinical trials published in the medical literature that have promising results in animal models of type 1 diabetes where stem cells are injected intravenously and it has established euglycemia in diabetic rats. Since stem cells are given intravenously to achieve a low concentration in the pancreas, I developed a novel technique of implanting the stem cells directly into the pancreas through the arterial blood supply. This article publishes a complete series of intrapancreatic autologous bone marrow-derived stem cell therapy for type 1 diabetes in humans which is reported for the first time in the medical literature.

## Materials and methods

### Study design

In the last 5 years, I treated 25 patients with type 1 diabetes with stem cell therapy, in which autologous bone marrow and omental fat-derived stem cells were implanted into the pancreas through the arteries supplying the pancreas. The age range in the therapy group was 10 years to 40 years. A group of 26 patients with type 1 diabetes taking the conventional treatment of insulin injections was put as a control group in the same period. Patients were distributed in both groups in a random way. Out of them, 22 patients (88%) had the therapy done once, and the remaining 3 patients (12%) had the therapy done twice with a gap of one year. The primary objective of the study was to implant a maximum number of stem cells into the pancreas. The secondary objective of the study was to check the safety of the intervention and to document any adverse events. The tertiary objective of the study was to evaluate the effects of the intervention on symptoms of type 1 diabetes, insulin requirement, blood sugar and HbA1c levels, C-peptide levels, and autoimmunity and weight changes.

### Inclusion criterion

Patients were selected based on World Medical Association Helsinki Declaration for Ethical Principles for medical research^4^ involving human subjects. Patients must have a confirmed diagnosis of type 1 diabetes, which is usually done through blood tests and clinical evaluation. Patients were diagnosed with type 1 diabetes^5^ clinically based on the following criterion. Age is an important consideration for the diagnosis of type 1 diabetes. Many patients had an early age at the onset of diabetes. Most of them presented as ketoacidosis with severe symptoms such as severe weight loss, extreme weakness, polyuria, and bed-wetting. The duration of the disease is an important consideration for stem cell therapy. Stem cell therapy is more effective in patients who have had type 1 diabetes of a shorter duration, as it is easier to regenerate the beta cells that have been recently damaged or destroyed. Patients who have had type 1 diabetes for a longer duration may not be suitable candidates for stem cell therapy, as the beta cells may be completely destroyed, making it difficult to regenerate them. All patients’ blood investigations were done, which showed high blood sugar levels of more than 200 mg/dl with high HbA1c levels. Almost all patients had a very low endogenous insulin production with a very low blood C-peptide level. Only a few patients had higher-than-normal anti-glutamic acid decarboxylase (GAD) antibody levels. It was objective evidence of autoimmunity. There are many tests described for the detection of autoimmunity in type 1 diabetes. In a Japanese study^6^, the most frequently detected autoantibodies are GAD autoantibodies (~80%) followed by IA2 autoantibodies (~60%), insulin autoantibodies (~55%) and ZnT8 autoantibodies (~50%). But since the anti-GAD antibody test was locally available and affordable, it was performed on all patients. All the patients were insulin dependent since the onset of the disease. All patients had a minimal follow-up period of at least 6 months after the initiation of the insulin therapy. Patients must be in good general health to undergo stem cell therapy. They should not have any other chronic illnesses or medical conditions that may interfere with the success of stem cell therapy. All patients selected had no altered endocrine status other than diabetes mellitus without any other debilitating disease conditions.

### Exclusion criteria

*Age*: Intrapancreatic stem cell therapy is generally not recommended for patients who are too young or too old. Children under the age of 10 may not be suitable candidates due to the potential risks associated with femoral arterial puncture. Similarly, older adults may not be suitable due to the decreased efficacy of stem cells in older patients. Hence, patients older than 50 years were excluded from the study. *Medical history*: Patients with a history of cancer, autoimmune disorders, or other serious medical conditions may not be suitable candidates for stem cell therapy. This is because the therapy can potentially exacerbate these conditions or increase the risk of complications. Those patients who had acute infections such as HIV/HBV/HCV (human immunodeficiency virus/hepatitis B virus/hepatitis C virus), malignancies, bleeding tendencies, renal failure, severe liver dysfunction, and other acute medical conditions such as respiratory infection and pyrexia were excluded from the study. *Pregnancy*: Stem cell therapy is not recommended for pregnant women. The potential risks to the fetus are unknown, and there is a risk of rejection of the stem cells by the mother’s immune system. *Blood sugar control*: Patients who have difficulty controlling their blood sugar levels may not be suitable candidates for stem cell therapy. This is because stem cells are most effective in patients with well-controlled blood sugar levels. *Allergies*: Patients with a history of severe allergies or allergic reactions may not be suitable candidates for stem cell therapy. This is because the therapy involves the use of biological materials, which can potentially trigger an allergic reaction. *Smoking*: Patients who smoke may not be suitable candidates for stem cell therapy. Smoking can decrease the effectiveness of stem cells and increase the risk of complications. *Medications*: Patients who are taking certain medications, such as immunosuppressants, may not be suitable candidates for stem cell therapy. These medications can interfere with the efficacy of stem cells or increase the risk of complications. Very few patients who were managed on diet and oral hypoglycemic drugs alone without insulin were also excluded from the study.

### Pre-intervention assessment

The pre-intervention assessment is critical in determining the appropriate stem cell therapy protocol, identifying potential risks and contraindications, and developing a personalized treatment plan that is tailored to the patient’s specific needs and medical history. It is an essential step in ensuring the safety and efficacy of stem cell therapy and maximizing its potential benefits for the patient. It is the process of evaluating a patient’s medical history, physical examination, and diagnostic tests to determine their eligibility and suitability for intrapancreatic stem cell therapy. The pre-intervention assessment includes a comprehensive evaluation of the patient’s medical history, including their current symptoms, medical conditions, past medical procedures, medications, and allergies. A physical examination is also performed to assess the patient’s general health and identify any abnormalities or underlying conditions that may affect the outcome of stem cell therapy. In addition, the patient may undergo a battery of diagnostic tests, including blood tests, imaging studies, and other specialized tests to determine the type and severity of the condition being treated, as well as the optimal stem cell therapy approach. All the patients underwent a thorough clinical examination with serological, biochemical, and hematological tests prior to the intervention. Blood sugar fasting and postprandial, C-peptide levels, anti-GAD antibody titer, glycosylated Hb, and weight of a patient with total insulin requirement in 24 h were the variables to be measured before the therapy and afterward at every 3 monthly intervals. Renal function tests such as BUN (blood urea nitrogen) and serum creatinine as done as patients along with HIV and HBSAG (hepatitis B surface antigen) tests. Special informed consent was taken from the parents of all the patients.

### Institutional ethical committee clearance^7^,^8^

A written permission was taken from the Institutional Ethical Committee (IEC) set at the district IMA (Indian Medical Association) level of the institution. An Institutional Committee for Stem Cell Research (IC-SCR) was established at the district level for peer review of stem cell research and its permission taken for the study. The guidelines set by the Indian Council of Medical Research (ICMR) were strictly followed^8^. They are as follows: Two stem cell therapy experts with a degree in regenerative medicine were attached to my institute, and the whole research program is conducted under their supervision. My institute got accredited by the National Accreditation Board of Hospitals (NABH).

### Patient symptomatology

In the therapy group, 5 patients (20%) presented with significant weakness, 18 patients (72%) had significant weight loss, 7 patients (28%) had polyuria, 6 patients (24%) had polyphagia, 3 patients had blurred vision with vision loss (12%), 4 patients (16%) had peripheral neuropathy, and 2 patients (8%) complained of erectile dysfunction. Three patients (12%) had presented with diabetic ketoacidosis with coma. Only 6 patients (24%) had autoimmunity with significantly raised anti-GAD antibody levels (Table 1).

**Table 1:** Patient data in the therapy group.

Sr. no	Date of therapy	Age (year)	Sex	History	Clinical features	Investigations before therapy	Insulin before therapy (IU) in 24 h	Source of stem cells used	Side effects of therapy	Clinical improvement 1-year after therapy	Units of insulin after therapy in 24 h					Investigations after therapy				
											6 months	1-year	2 years	3 years	4 years	6 months	1 year	2 years	3 years	4 years
1	04/8/2017	20	M	KCO type 1 diabetes for 2 years	Weakness, Peripheral neuropathy Weight 51 kg	Hba1c 9.1 Blood Sugar F 210 Blood Sugar pp 320 C-peptide 0.05 Anti-GAD antibody 23.7	45	Omental fat	Skin rash	Weight gain of 8 kg in 1-year, peripheral neuropathy symptoms disappeared completely	30	20	20	20	20	Hba1c 8.1 Blood Sugar F 186 Blood Sugar pp 210	Hba1c 7.3 Blood Sugar F 156 Blood Sugar pp 185 C-peptide 0.15 Anti-GAD antibody 11.6	Hba1c 7.4 Blood Sugar F 162 Blood Sugar pp 190	Hba1c 7.3 Blood Sugar F 152 Blood Sugar pp 175	Hba1c 7.4 Blood Sugar F 160 Blood Sugar pp 185
2	13/10/2017	15	M	KCO type 1 diabetes for 1-year	Weakness, Bed-wetting, Peripheral neuropathy, Weight 53 kg	Hba1c 12.4 Blood Sugar F 310 Blood Sugar pp 452 C-peptide 0.01 Anti-GAD antibody 13.7	70	Omental fat	Nil	Weight gain of 6 kg in 1-year, peripheral neuropathy symptoms disappeared completely, bed-wetting completely stopped	50	35	35	35	35	Hba1c 8.3 Blood Sugar F 185 Blood Sugar pp 258 C-peptide 0.15	Hba1c 8.1 Blood Sugar F 180 Blood Sugar pp 252 C-peptide 0.15 Anti-GAD antibody 13.7	Hba1c 8.2 Blood Sugar F 175 Blood Sugar pp 252	Hba1c 8.1 Blood Sugar F 170 Blood Sugar pp 248	Hba1c 8.2 Blood Sugar F 175 Blood Sugar pp 262
3	29/11/2017	10	M	KCO type 1 diabetes for 6 months	HO of diabetic ketoacidosis with coma, polydipsia, Weight 21 kg	Hba1c 9.5 Blood Sugar F 226 Blood Sugar pp 358 C-peptide 0.5 Anti-GAD antibody 5.47	12	Omental fat	Pain in abdomen	Weight gain of 5 kg in 1-year, improved energy, polydipsia disappeared completely	6	0	0	0	0	Hba1c 5.3 Blood Sugar F 91 Blood Sugar pp 98 C-peptide 0.58	Hba1c 5.0 Blood Sugar F 73 Blood Sugar pp 78 C-peptide 0.98	Hba1c 5.1 Blood Sugar F 89 Blood Sugar pp 92	Hba1c 5.2 Blood Sugar F 94 Blood Sugar pp 98	Hba1c 5.0 Blood Sugar F 78 Blood Sugar pp 82
4	25/08/2018	20	M	KCO type 1 diabetes for 1-year	HO extreme weight loss and polyuria, erectile dysfunction, Weight 37 kg	Hba1c 14 Blood Sugar F 392 Blood Sugar pp 458 C-peptide 0.1 Anti-GAD antibody 5.47	55	Bone marrow	Nil	Weight gain of 11 kg in 1-year, improved energy, improvement in erectile dysfunction	25	25	25	25	NA	Hba1c 7.8 Blood Sugar F 201 Blood Sugar pp 213 C-peptide 0.3	Hba1c 7.5 Blood Sugar F 193 Blood Sugar pp 203 C-peptide 0.42	Hba1c 7.3 Blood Sugar F 186 Blood Sugar pp 201	Hba1c 7.4 Blood Sugar F 176 Blood Sugar pp 198	NA
5	10/12/2018	27	M	KCO type 1 diabetes for 5 years	HO extreme weight loss and polyuria, Weight 68 kg	Hba1c 11.8 Blood Sugar F 250 Blood Sugar pp 358 C-peptide 0.05 Anti-GAD antibody 3.58	40	Bone marrow	Skin rash	Weight gain of 5 kg in 1-year, no polyuria	25	20	20	20	NA	Hba1c 7.5 Blood Sugar F 169 Blood Sugar pp 230 C-peptide 0.10	Hba1c 6.5 Blood Sugar F 140 Blood Sugar pp 210 C-peptide 0.15	Hba1c 6.8 Blood Sugar F 169 Blood Sugar pp 230	Hba1c 7.0 Blood Sugar F 154 Blood Sugar pp 215	NA
6	11/12/2018	21	F	KCO type 1 diabetes for 15 years	HO weight loss and polyuria, weakness, Peripheral neuropathy, Weight 58 kg	Hba1c 11.5 Blood Sugar F 250 Blood Sugar pp 350 C-peptide 0.22 Anti-GAD antibody 5.47	50	Bone marrow	Nil	Weight gain of 8 kg in 1-year, improved energy, no polyuria	25	25	25	25	NA	Hba1c 7.3 Blood Sugar F 150 Blood Sugar pp 220 C-peptide 0.36	Hba1c 7.0 Blood Sugar F 130 Blood Sugar pp 190 C-peptide 0.45	Hba1c 7.3 Blood Sugar F 163 Blood Sugar pp 201	Hba1c 7.1 Blood Sugar F 143 Blood Sugar pp 198	NA
7	19/02/2019	22	M	KCO type 1 diabetes for 2 years	HO severe weight loss and polyuria, weakness, Peripheral neuropathy, Weight 42 kg	Hba1c 15 Blood Sugar F 448 Blood Sugar pp 600 C-peptide 1.42 Anti-GAD antibody 3.35	100	Bone marrow	Femoral hematoma	Weight gain of 7 kg in 1-year, improved energy, no polyuria, neuropathy symptoms reduced by 50%	75	50	50	NA	NA	Hba1c 11 Blood Sugar F 269 Blood Sugar pp 302 C-peptide 1.42	Hba1c 9.2 Blood Sugar F 212 Blood Sugar pp 236 C-peptide 2.42	Hba1c 9.1 Blood Sugar F 202 Blood Sugar pp 216 C-peptide 1.42	NA	NA
8	12/03/2019	11	M	KCO type 1 diabetes for 5 months	HO weight loss and polyuria, HO ketoacidosis, Weight 23 kg	Hba1c 14 Blood Sugar F 355 Blood Sugar pp 550 C-peptide 0.23 Anti-GAD antibody 29.77	10	Bone marrow	Mild nausea	Weight gain of 3 kg in 1-year, improved energy	5	5	5	5	NA	Hba1c 8.7 Blood Sugar F 203 Blood Sugar pp 248 C-peptide 0.23	Hba1c 8.3 Blood Sugar F 192 Blood Sugar pp 230 C-peptide 0.56 Anti-GAD antibody 19.2	Hba1c 8.1 Blood Sugar F 183 Blood Sugar pp 215	Hba1c 8.3 Blood Sugar F 192 Blood Sugar pp 223	NA
9	06/05/2019	19	M	KCO type 1 diabetes for 7 years	HO weight loss and polyphagia Weight 50 kg	Hba1c 5.95 Blood Sugar F 126 Blood Sugar pp 210 C-peptide 0.52 Anti-GAD antibody 5.2	42	Bone marrow	Nil	Weight gain of 8 kg in 1-year, improved energy, no polyphagia	25	15	15	NA	NA	Hba1c 4.8 Blood Sugar F 96 Blood Sugar pp 154 C-peptide 0.55	Hba1c 4.4 Blood Sugar F 90 Blood Sugar pp 140 C-peptide 0.55	Hba1c 4.0 Blood Sugar F 80 Blood Sugar pp 110 C-peptide 0.75	NA	NA
10	24/06/2019	11	M	KCO type 1 diabetes for 1-year	HO weight loss and polyphagia Weight 38 kg	Hba1c 9.4 Blood Sugar F 239 Blood Sugar pp 378 C-peptide 0.23 Anti-GAD antibody 98	28	Bone marrow	Pain in abdomen	Weight gain of 8 kg in 1-year, improved energy	30	20	20	NA	NA	Hba1c 7.4 Blood Sugar F 166 Blood Sugar pp 248 C-peptide 0.22	Hba1c 6.62 Blood Sugar F 143 Blood Sugar pp 228 C-peptide 0.42 Anti-GAD antibody 55	Hba1c 6.58 Blood Sugar F 141 Blood Sugar pp 221	NA	NA
11	30/08/2019	21	M	KCO type 1 diabetes for 2 years	HO stem cell therapy 1-year ago, Weight 50 kg	Hba1c 7.5 Blood Sugar F 193 Blood Sugar pp 203 C-peptide 0.42	25	Bone marrow	Nil	Weight gain of 3 kg in 1-year, improved energy	10	0	0	NA	NA	Hba1c 6.1 Blood Sugar F 128 Blood Sugar pp 168 C-peptide 0.35	Hba1c 5.5 Blood Sugar F 111 Blood Sugar pp 153 C-peptide 0.65	Hba1c 5.8 Blood Sugar F 121 Blood Sugar pp 161	NA	NA
12	04/09/2019	14	M	KCO type 1 diabetes for 7 years	HO weight loss and polyphagia, bed-wetting, Weight 32 kg	Hba1c 10.5 Blood Sugar F 238 Blood Sugar pp 339 C-peptide 0.02 Anti-GAD antibody 42.2	45	Bone marrow	Pain in abdomen	Weight gain of 5 kg in 1-year, improved energy, no bed-wetting	45	25	25	NA	NA	Hba1c 8.9 Blood Sugar F 201 Blood Sugar pp 231 C-peptide 0.1	Hba1c 8.5 Blood Sugar F 195 Blood Sugar pp 211 C-peptide 0.3 Anti-GAD antibody 18	Hba1c 8.4 Blood Sugar F 189 Blood Sugar pp 209	NA	NA
13	06/09/2019	25	M	KCO type 1 diabetes for 7 years	HO weight loss and polyphagia, erectile dysfunction Weight 52 kg	Hba1c 9.4 Blood Sugar F 205 Blood Sugar pp 278 C-peptide 0.05 Anti-GAD antibody 17.5	50	Bone marrow	Nil	Weight gain of 8 kg in 1-year, improved energy, improvement in erectile dysfunction, no polyphagia	30	20	20	NA	NA	Hba1c 8.1 Blood Sugar F 167 Blood Sugar pp 215 C-peptide 0.08	Hba1c 7.8 Blood Sugar F 158 Blood Sugar pp 205 C-peptide 0.1	Hba1c 8.0 Blood Sugar F 178 Blood Sugar pp 215	NA	NA
14	14/12/2019	10	M	KCO type 1 diabetes for 6 months	HO weight loss and polyuria Weight 38 kg	Hba1c 11.4 Blood Sugar F 250 Blood Sugar pp 410 C-peptide 0.15 Anti-GAD antibody 2000	18	Bone marrow	Mild Skin rash	Weight gain of 6 kg in 1-year, improved energy, no polyuria	10	15	15	NA	NA	Hba1c 9.5 Blood Sugar F 226 Blood Sugar pp 221 C-peptide 0.15	Hba1c 8.9 Blood Sugar F 209 Blood Sugar pp 210 C-peptide 0.35 Anti-GAD antibody 950	Hba1c 9.1 Blood Sugar F 215 Blood Sugar pp 231	NA	NA
15	23/12/2019	38	M	KCO type 1 diabetes for 5 years	HO weight loss and polyphagia, Weight 58 kg	Hba1c 10.4 Blood Sugar F 252 Blood Sugar pp 358 C-peptide 0.05 Anti-GAD antibody 1.3	50	Bone marrow	Nil	Weight gain of 5 kg in 1-year, improved energy, no polyuria	40	25	25	NA	NA	Hba1c 8.8 Blood Sugar F 206 Blood Sugar pp 238 C-peptide 0.8	Hba1c 8.4 Blood Sugar F194 Blood Sugar pp 221 C-peptide 0.15	Hba1c 8.6 Blood Sugar F198 Blood Sugar pp 228	NA	NA
16	28/12/2019	29	M	KCO type 1 diabetes for 8 years	HO weight loss and polyuria, blurred vision, Weight 55 kg	Hba1c 11.6 Blood Sugar F 286 Blood Sugar pp 368 C-peptide 0.15 Anti-GAD antibody 17	45	Bone marrow	Pain in abdomen, inguinal ecchymosis 3 cm	Weight gain of 5 kg in 1-year, improved energy, no polyuria, improved vision	25	25	25	NA	NA	Hba1c 9.1 Blood Sugar F 214 Blood Sugar pp 248 C-peptide 0.15	Hba1c 8.6 Blood Sugar F 200 Blood Sugar pp 231 C-peptide 0.35	Hba1c 8.5 Blood Sugar F 196 Blood Sugar pp 221	NA	NA
17	26/02/2020	10	M	KCO type 1 diabetes for 6 months	HO of diabetic ketoacidosis with coma, HO weakness and polyuria Weight 24 kg	Hba1c 10.8 Blood Sugar F 235 Blood Sugar pp 358 C-peptide 0.14 Anti-GAD antibody 2000	20	Omental fat	Nil	Weight gain of 3 kg in 1-year, improved energy	10	15	NA	NA	NA	Hba1c 10.6 Blood Sugar F 286 Blood Sugar pp 395 C-peptide 0.12	Hba1c 9.8 Blood Sugar F 215 Blood Sugar pp 315 C-peptide 0.24 Anti-GAD antibody 830	Hba1c 10.5 Blood Sugar F 205 Blood Sugar pp 305	NA	NA
18	14/10/2020	40	M	KCO type 1 diabetes for 15 years	HO weight loss, lack of energy and polyphagia Weight 50 kg	Hba1c 7.2 Blood Sugar F 128 Blood Sugar pp 174 C-peptide 0.3 Anti-GAD antibody 12.67	38	Bone marrow	Mild nausea	Weight gain of 9 kg in 1-year, Improved energy, no polyphagia	25	15	NA	NA	NA	Hba1c 7.1 Blood Sugar F 157 Blood Sugar pp 158 C-peptide 0.35	Hba1c 6.2 Blood Sugar F 101 Blood Sugar pp 138 C-peptide 0.65 Anti-GAD antibody 12.67	Hba1c 6.0 Blood Sugar F 98 Blood Sugar pp 128	NA	NA
19	19/10/2020	17	F	KCO type 1 diabetes for 2 years	HO weight loss, lack of energy Weight 48 kg	Hba1c 16.8 Blood Sugar F 456 Blood Sugar pp 533 C-peptide 0.18 Anti-GAD antibody 7.4	45	Bone marrow	Inguinal ecchymosis 5 cm	Weight gain of 10 kg in 1-year, improved energy	25	25	NA	NA	NA	Hba1c 10.9 Blood Sugar F 209 Blood Sugar pp 258 C-peptide 0.18	Hba1c 8.5 Blood Sugar F 158 Blood Sugar pp 238 C-peptide 0.28	Hba1c 8.2 Blood Sugar F 128 Blood Sugar pp 218	NA	NA
20	12/01/2021	24	M	KCO type 1 diabetes for 3 years	HO weight loss, HO vision loss, blurred vision, Weight 58 kg	Hba1c 12.2 Blood Sugar F 195 Blood Sugar pp 394 C-peptide 0.25 Anti-GAD antibody 1.2	36	Bone marrow	Nil	Weight gain of 8 kg in 6 months, improvement in vision	18	0	NA	NA	NA	Hba1c 8.8 Blood Sugar F 175 Blood Sugar pp 255 C-peptide 0.46	Hba1c 8.5 Blood Sugar F 150 Blood Sugar pp 245 C-peptide 0.98	NA	NA	NA
21	12/05/2021	13	M	KCO type 1 diabetes for 12 years	HO of diabetic ketoacidosis with coma, Dwarfism, inability to walk due to hip arthritis Weight 26 kg	Hba1c 12.95 Blood Sugar F 312 Blood Sugar pp 394 C-peptide 0.25 Anti-GAD antibody 9.14	20	Omental fat	Pain in abdomen	Weight gain of 2 kg in 6 months	10	10	NA	NA	NA	Hba1c 9.3 Blood Sugar F 155 Blood Sugar pp 210 C-peptide 0.25	Hba1c 9.1 Blood Sugar F 145 Blood Sugar pp 198 C-peptide 0.25	NA	NA	NA
22	18/05/2021	37	M	KCO type 1 diabetes for 2 years	HO weight loss, HO vision loss, Weight 48 kg	Hba1c 12.4 Blood Sugar F 295 Blood Sugar pp 380 C-peptide 0.02 Anti-GAD antibody 9.14	36	Bone marrow	Nil	Weight gain of 10 kg in 6 months	25	25	NA	NA	NA	Hba1c 8.4 Blood Sugar F 195 Blood Sugar pp 210 C-peptide 0.15	Hba1c 8.1 Blood Sugar F 175 Blood Sugar pp 195 C-peptide 0.2	NA	NA	NA
23	20/11/2021	16	M	KCO type 1 diabetes for 9 years	HO weight loss and polyphagia, bed-wetting, Weight 37 kg	Hba1c 8.4 Blood Sugar F 178 Blood Sugar pp 212 C-peptide 0.05 Anti-GAD antibody 42.2	25	Omental fat	Inguinal ecchymosis 2 cm	Weight gain of 3 kg in 6 months, improved energy, no bed-wetting	25	0	NA	NA	NA	Hba1c 8.4 Blood Sugar F 201 Blood Sugar pp 231 C-peptide 0.3	Hba1c 7.8 Blood Sugar F 165 Blood Sugar pp 198 C-peptide 0.5	NA	NA	NA
24	11/02/2022	38	M	KCO type 1 diabetes for 3 years	HO weight loss, HO vision loss, Weight 58 kg	Hba1c 8.1 Blood Sugar F 175 Blood Sugar pp 195 C-peptide 0.2 Anti-GAD antibody 9.14	15	Omental fat	Nil	Weight gain of 2 kg in 6 months	15	0	NA	NA	NA	Hba1c 8.1 Blood Sugar F 175 Blood Sugar pp 195 C-peptide 0.2	Hba1c 7.8 Blood Sugar F 155 Blood Sugar pp 175 C-peptide 0.4	NA	NA	NA
25	02/06/2022	11	M	KCO type 1 diabetes for 1 year	HO weight loss, polyuria, Weight 28 kg	Hba1c 6.7 Blood Sugar F 112 Blood Sugar pp 189 C-peptide 0.44 Anti-GAD antibody 6.55	24	Omental fat	Skin rash	Weight gain of 3 kg in 6 months	0	NA	NA	NA	NA	Hba1c 4.8 Blood Sugar F 89 Blood Sugar pp 126 C-peptide 0.78	NA	NA	NA	NA

Anti-GAD, anti-glutamic acid decarboxylase; HO, history of; KCO, known case of; NA, not applicable.

### Source of stem cells

The two main sources of stem cells in the human body are bone marrow and adipose tissue. In this series, in 8 patients (32%), omental fat was used as a source of stem cells and in the remaining 17 patients (68%), bone marrow was used (Table 1). Both sources are used in this series due to a variety of reasons which are as follows. Many patients with type 1 diabetes are cachectic with poor weight and very little fat in their bodies. But they have good hemoglobin. Such patients were chosen for bone marrow-derived stem cells, the bone marrow aspirate concentrate (BAMC). The only contraindication for the above option was for patients with low hemoglobin and bone marrow disorders. The second option is adipose tissue, the adipose-derived stem cells (ADSC). Adipose tissue can be harvested by liposuction from abdominal and thigh fat. I used the omentum as the source for stem cells which is already been reported by me for the first time in the medical literature^9^. The omentun has very high-quality multipotent stem cells, which are not found in other adipose tissues in the body. Literature shows that there are various cells in the omentum with stem cell roles. Experimental models suggest that omentum contains pluripotent stem cells, which can differentiate into nerve cells, adipocytes, and hepatocytes^10,11^. Bone marrow has far less mesenchymal stem cells than adipose tissue. But many studies^12^ have shown that stem cells from both sources have good regenerative effects. I found both methods almost equally effective in this series. But we need a separate comparative study to find out more details about it.

### Separation protocol for ADSC^13^

Overall, the separation protocol for ADSCs involves a series of steps to isolate the cells from adipose tissue and remove any contaminants. The protocol can be modified based on the specific needs of the therapy, but the general steps remain the same. A small umbilical incision is taken and the omentum is delivered out of the umbilical incision. The vessels supplying the omentin are ligated, and the majority of omentun is excised to be used as a source for stem cells. The omental fat is finely chopped and put into four test tubes. An equal quantity of Hanks’ Balanced Salt Solution (HBSS) is poured over it, centrifuged for 10 min and the supernatant is discarded. This removes the greater majority of contaminating red blood cells.

#### Enzymatic digestion of the lipoaspirate^13^

Sterile collagenase type 1 solution is prepared in HBSS and an equal volume to that of the adipose tissue is poured over the adipose tissue. This solution is added with antibiotics such as streptomycin and antifungal agents like amphoteric B to avoid bacterial and fungal contamination. The test tubes are now kept in an incubator for enzymatic digestion for 12–24 h.

#### Isolation of ADSCs

The digested collagenase–adipose mixture is placed in the biosafety cabinet. It has four layers from above downward. The upper first layer of oil is due to the lysis of mature adipocytes, a middle second layer of adipose tissue, a third layer of liquid infranatant containing saline and contaminating cell types such as red blood cells (RBCs), and the lowest fourth layer at the bottom of the test tube called stromal vascular fraction (SVF) containing stem cells. The top three layers are pipetted out and discarded, keeping only SVF. Meanwhile, 50 ml blood of the same patient is collected in a heparinized syringe and centrifuged to get the serum. The patient’s serum is poured over the SVF and the sample is incubated at 37°C for 30 min and centrifuged at 1800*g* for 10 min. The serum is discarded, and the SVF (Stromal Vascular Fraction) is washed with HBSS and filtered by a 100 μm mesh filter and diluted in HBSS, and collected in 10 ml syringes for clinical use.

### Technique of bone marrow harvesting^14^

The procedure is conducted with total aseptic precautions in a modular operation theater with lamellar airflow. General anesthesia was given to patients below 18 years of age. Whereas patients above 18 years, spinal anesthesia was given. A Jamshidi bone marrow aspiration needle was attached to a disposable 50 ml syringe with heparin sodium as anticoagulant and 100-150 ml marrow was aspirated from the posterior inferior iliac spine. If bone marrow was not adequate, the anterior superior iliac spine and iliac crest were the next best options.

### Separation protocol for isolation of BMAC^15^

Twenty-five ml bone marrow is collected into a heparinized syringe of 50 ml and diluted with an equal quantity of Dulbecco’s Phosphate Buffered Saline with 2% Fetal Bovine Serum (PBS+2% FBS) (Sigma-Aldrich, USA). Disposable centrifuge tubes of 50 ml capacity are prepared and the diluted bone marrow is poured over an equal quantity of density gradient such as Lymphoprep (Stemcell Technologies, Vancouver, Canada) with a density of 1.077 g/ml. The tubes are centrifuged at 800*g* for 20 min at room temperature (15–25°C) with brakes off. The tubes have a buffy coat in the middle containing mononuclear cells stem cells. The buffy coat is aspirated and kept ready in 10 ml syringes for infusion. About 30–50 ml BMAC was isolated from each patient.

### Stem cell characterization^16^

Stem cell characterization refers to the process of identifying and describing the properties and behavior of stem cells. Stem cell characterization typically involves a variety of techniques to assess the stem cell’s morphology, gene expression, and functionality. Stem cell characterization is essential for understanding the properties and behavior of stem cells and for developing effective strategies for their therapeutic use. By characterizing stem cells, researchers can identify the best sources of stem cells for different applications and optimize their differentiation protocols to generate specific cell types for regenerative medicine.

Some of the most common techniques used in stem cell characterization include Morphological analysis, Surface marker analysis, Gene expression analysis, epigenetic analysis, etc. Most of the above-mentioned characterization methods were not necessary for this study, hence only a limited characterization was done as described below.

A quick study was performed at the level of my institution by cytology to check for the viability of stem cells, numbers, and bacterial contamination before implanting. A sample of harvested stem cells was sent for cytology to measure stem cell viability by Trypan blue and to measure stem cell count, and to look for bacterial contamination at a histopathology center out of the institution as well for a third-party confirmation. The average viability count of harvested stem cells was found to be 98.3%. The average numbers of cells harvested were 8.86×10^7^. Cell media companies like Sigma-Aldrich and Thermo Fisher Scientific have already established FDA-approved protocols for the separation and isolation of stem cells from bone marrow and fat. These same protocols and methods, and reagents were used in the study. Hence only a limited stem cell characterization, as described above, was performed to reduce the cost and save time. Also, the stem cells were freshly harvested and implanted without culture and any genetic modification. Hence, the costly immune cytochemistry tests were not necessary. The above method comes under the ‘Minimal intervention category’ as classified by the Indian Council of Medical Research (ICMR), which is excluded by the government of India under the tag of drugs, and hence is permitted to practice by clinicians in India without a clinical trial. Stem cells of specific types, such as hemopoietic and mesenchymal cells, can be further separated from BMAC and fat. But both have regenerated properties, and such protocol is complex and costly, hence it was not performed^16^.

### The technique of cannulating the pancreatic artery and splenic artery by femoral arterial puncture is as follows

The patient is taken to a catheterization laboratory by ambulance. An infusion of 20 mg/kg methylprednisolone in 100 mlL normal saline started intravenously over 1 h before transplanting stem cells^17^. It prevents post-stem cell transplantation engraftment syndrome. Engraftment syndrome encompasses a continuum of peri-engraftment complications after autologous stem cell transplantation.

**Figure 1:**
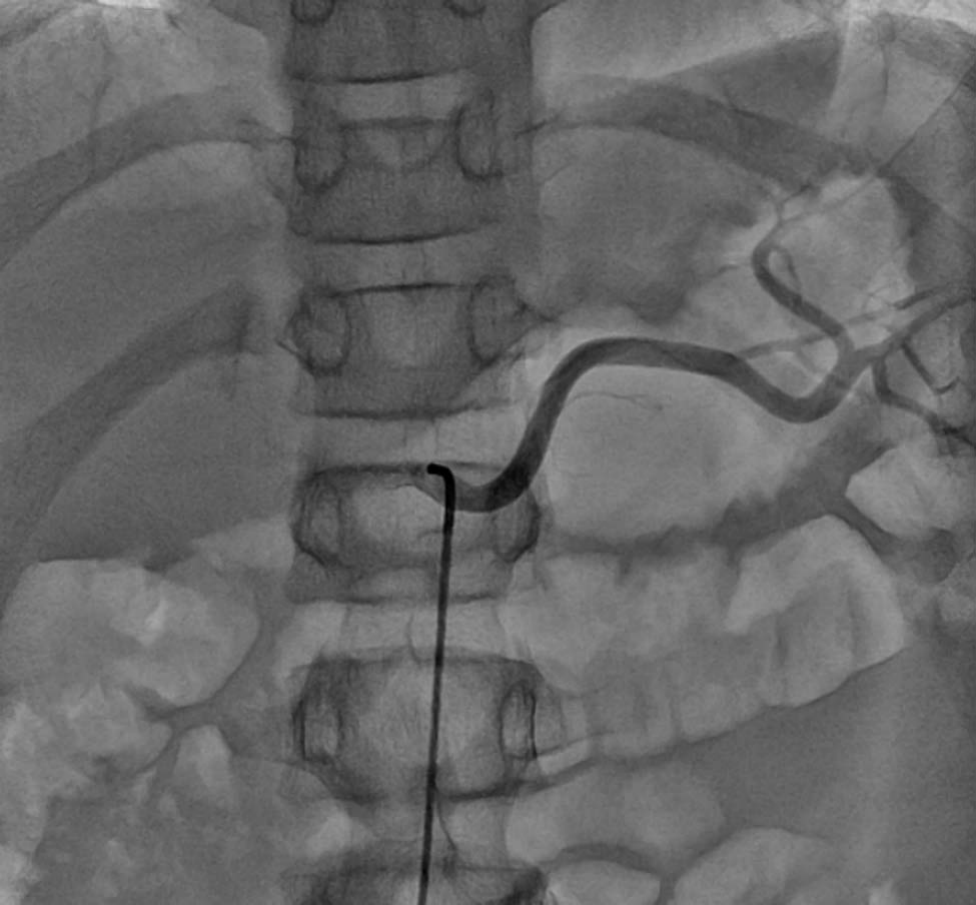
Angiogram showing the splenic artery.

**Figure 2:**
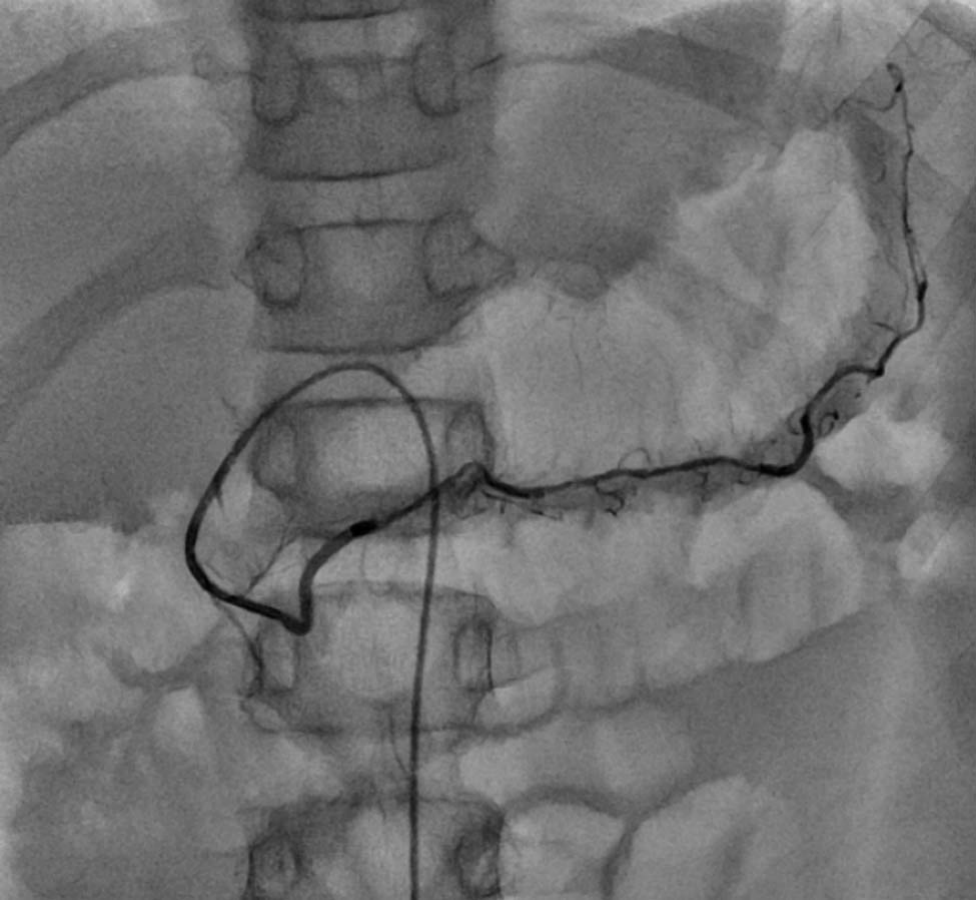
Angiogram showing the main pancreatic artery.

The right femoral area is prepared and draped. The intervention was carried out under fluoroscopic guidance by the interventional radiologist with a digital subtraction angiography technique. An adequate amount of 2% Lignocaine was injected into the femoral area as local anesthesia. The femoral artery is cannulated with modified Selinger’s technique which is as follows^18^. With the no. 18 needle, the femoral artery is punctured. A guide wire of size 0.35 in is inserted into the femoral artery, and the needle is removed. A femoral sheath of 6 F was inserted into the femoral artery over the guide wire, and the guide wire was removed. A 5 F arterial catheter was selectively navigated through a trans-femoral route into the aorta and radioactive dye was injected to look for the celiac artery. A guide wire passed through the catheter and guided into celiac artery and further into the splenic artery. The catheter passed over the guide wire into the middle part of the splenic artery (Fig. 1). Fifty percent quantity of stem cells were injected slowly over 15 min here (Video 1, Supplemental Digital Content 1, http://links.lww.com/MS9/A205) to reach the tail part of pancreases where 80% islets of Langerhans are located. The splenic artery also supplies to the body of pancreas through dorsal and greater pancreatic arteries. The catheter was withdrawn and a guide wire guided into the gastroduodenal artery. It divides into anterior and posterior pancreaticoduodenal arteries, which supply the head of the pancreas (Fig. 2). Remaining 50% quantity of stem cells were injected here to reach the head and body of the pancreas. The average number of cells transplanted were 8.86×10^7^.

Some patients had anomalies of blood supply to the pancreas^19^, which were as follows. Patient no. 13,22,23 had the celiac trunk with acute angle to the aorta; hence, the catheter could not be passed beyond it. In these patients, stem cells were injected into celiac trunk as further catheterization was not possible.

### Post-stem cell transplantation care

The adverse effects of the therapy were monitored by examining the patients regularly. Vital parameters of patients, such as pulse, blood pressure, and respiration, were regularly monitored. A daily chart of blood sugar fasting and postprandial was maintained by taking readings regularly with a glucometer.

### Outcome measures

The outcome measures were used to monitor any major or minor adverse events throughout the entire duration of follow-up to assess the safety of the intervention. Regular counseling of patients was performed to know the probable adverse events during follow-up period. During the hospital stay, recording of adverse events was done by a health professional in the hospital. After discharge, patients were followed up by the parents and patients' diabetologists in their local area, and the reports and events were recorded and reported by them through e-mail and telephone.

### Monitoring procedure-related adverse events^20^

Hematoma at the bone marrow aspiration site and at the femoral puncture site, infection, pain, and vomiting are the complications described in medical literature for the above procedure. Over 5–7 days after the intervention, the acute procedural adverse events associated with cell aspiration and injection were monitored. Body temperature, blood pressure, respiratory rate, blood sugar fasting, postprandial, and heart rate were recorded at regular intervals. Daily, the aspiration and transplantation sites were examined for pain, bleeding, and signs of infection. Anesthesia complications such as headache, nausea, and vomiting were regularly checked. All the acute procedural adverse events were treated prior to the discharge of the patients from the hospital.

### Monitoring cellular transplantation-related adverse events^21^

Skin rash, fever, an immune reaction against implanted cells, and vomiting are described in the medical literature as adverse reactions to cell transplantation. Patient’s signs and symptoms of any allergic reaction were monitored at regular intervals during their stay in the hospital. Major and minor adverse events were monitored on a long-term basis to establish the safety of stem cell transplantation.

### Monitoring the effects after intervention

Postoperatively, the sugar levels were regularly monitored daily by a glucometer, and the dose of insulin injections was adjusted accordingly. The variables to be compared were Blood sugar fasting and postprandial, Anti-GAD antibody titer, Glycosylated Hb and C-peptide levels, along with the patient’s weight and total daily insulin requirement. At every three monthly intervals, these parameters were checked before and after the therapy (Table 1).

### Statistical analysis^22^

The data for all the patients were recorded and analyzed. Mean age in years at the time of diagnosis, mean age in years at the time of intervention, and mean time duration in months at which the patients were followed up were calculated. The pre-intervention and post-intervention scores of *P* value were compared for the statistical level of significance (*T*-test) to check if it was more than or less than 0.01 and 0.05. The variables to be compared were the blood sugar fasting and postprandial, Glycosylated Hb, C-peptide levels, Anti-GAD antibody titer, and weight of the patient, along with total daily insulin requirement before and after one year of the therapy in both groups. Percentage analysis was performed for all of them. The total analysis was done as a *t*-test in the Microsoft Excel program.

## Results

The follow-up range was a minimum of 6 months to a maximum of 5 years (Table 1). It took 3 months to see the results. The parents of the patients daily monitored blood sugar by a glucometer and the dose of insulin was adjusted accordingly by the patient’s physician and a daily chart was maintained at home. Blood sugar fasting and postprandial, Glycosylated Hb, C-peptide levels, Anti-GAD antibody titer, along with patient’s weight and total daily insulin requirement, were the variables to be compared. These parameters were checked before the therapy and afterward at every 3 monthly intervals. At the end of one year after therapy, the clinical results were completely expressed. Hence, at the end of one year from the therapy date, the above variables were compared. Due to a variety of reasons, such as lack of affordability, lack of motivation, and lack of availability in their local area of residence, some patients did not send investigation reports at 3 monthly intervals.

Weight gain is an important prognostic indicator in type 1 diabetes. Weight just before therapy and one year after therapy was measured in both groups (Chart A) In the therapy group, the average weight gain after therapy was about 14% which came as statistically highly significant with *P*<0.01 (Charts A, B). In the control group, the average weight gain was just 2.51% and the difference was not statistically significant *P*>0.05 (Chart C).

**Figure:**
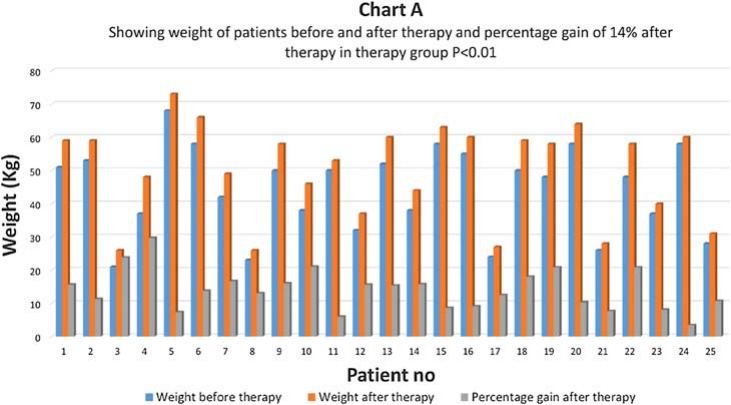


**Figure:**
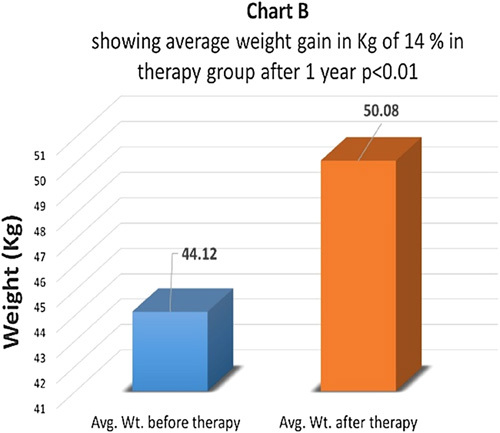


**Figure:**
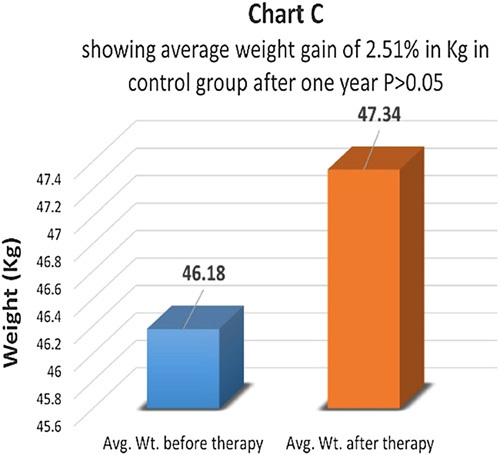


At the end of one year after therapy, in the therapy group, patients’ average daily insulin requirements before and after therapy were compared (Chart D). It dropped by 59.14% and the difference came as statistically highly significant with *P*<0.01 (Chart D). Six (24%) patients went off insulin and are free of insulin with a follow-up of 6 months to 5 years after therapy. Out of them, three patients (12%) had the therapy done once and the remaining three patients (12%) had the therapy done twice with a gap of one year. In the control group, no patient went off insulin. The average daily insulin dropped by 11.45% and the difference was not significant statistically *P*>0.05 (Chart F). In 65.94% of patients, daily insulin requirements were the same and in 34.06% of patients daily insulin requirements were higher than before.

**Figure:**
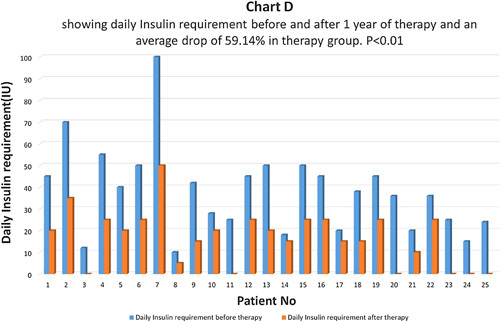


**Figure:**
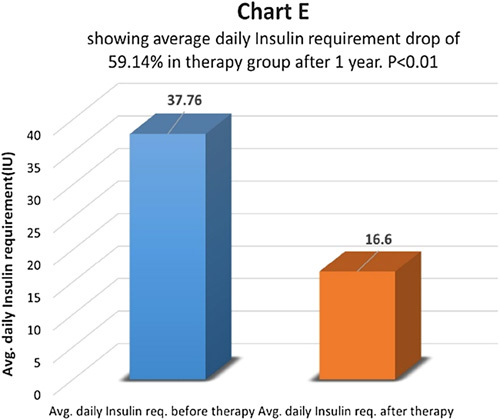


**Figure:**
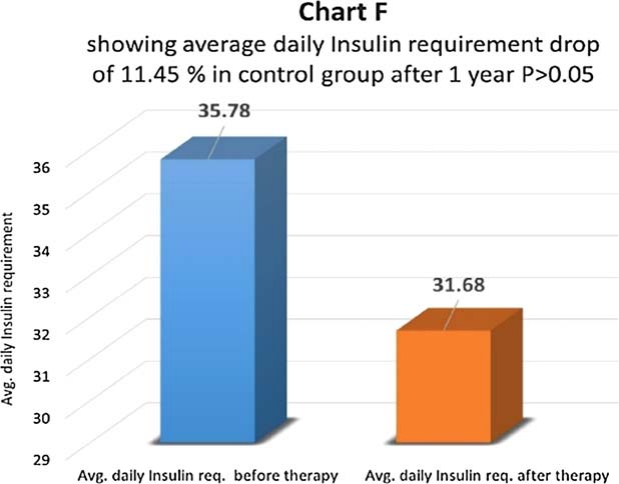


HbA1c levels before and after therapy were compared in a therapy group (Chart G). There was an average drop of 28.14% in the therapy group and the difference came as statistically highly significant with *P*<0.01 (Chart H). The control group had an average drop of only 8.2% in HbA1c levels and the difference was not statistically significant *P*>0.05 (Chart I). 72.6% of patients had the same HbA1c levels and 27.4% of patients had levels higher than before in the control group.

**Figure:**
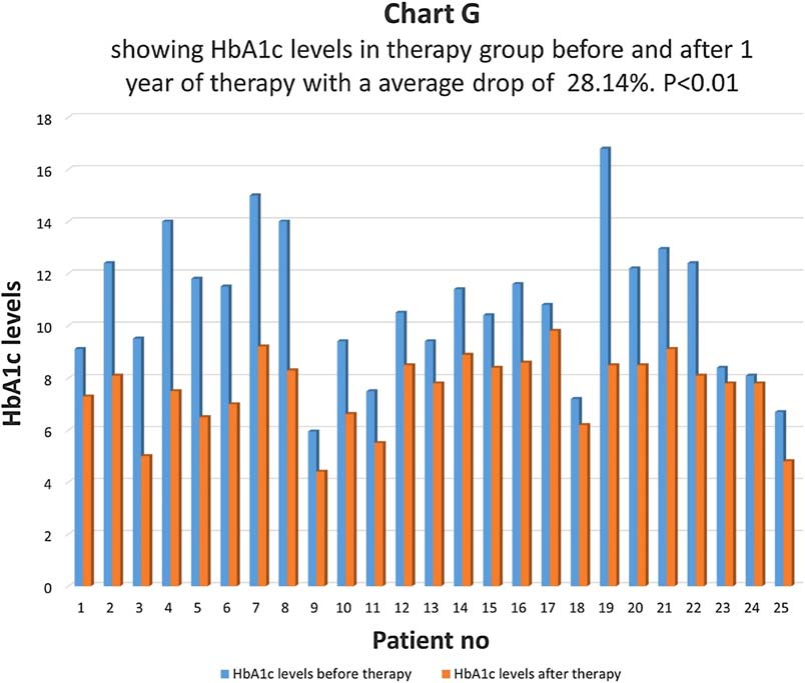


**Figure:**
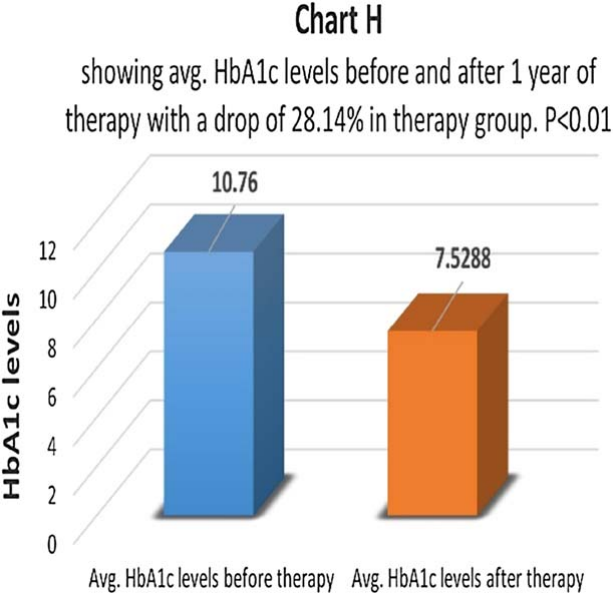


**Figure:**
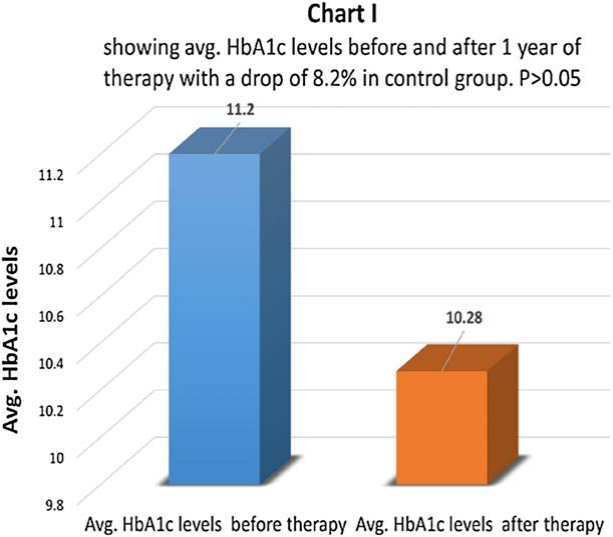


Fasting blood sugar levels were compared before and one year after therapy in the therapy group (Chart J). There was an average drop of 33.86% in fasting blood sugar in the therapy group and the difference was statistically highly significant with *P*<0.01 (Chart K). In the control group, there was an average drop of 11.62% in fasting blood sugar levels and the difference was not statistically significant, *P*>0.05 (Chart L). In the control group, 69.8% of patients had the same fasting blood sugar, and 30.2% of patients had a higher postprandial blood sugar level.

**Figure:**
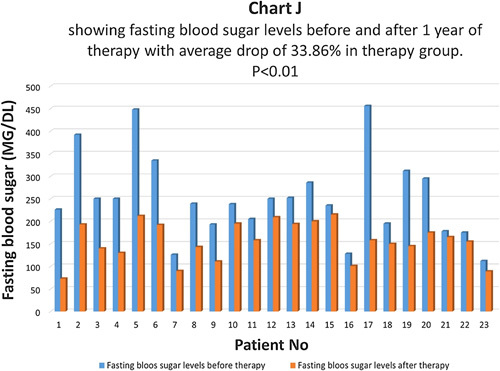


**Figure:**
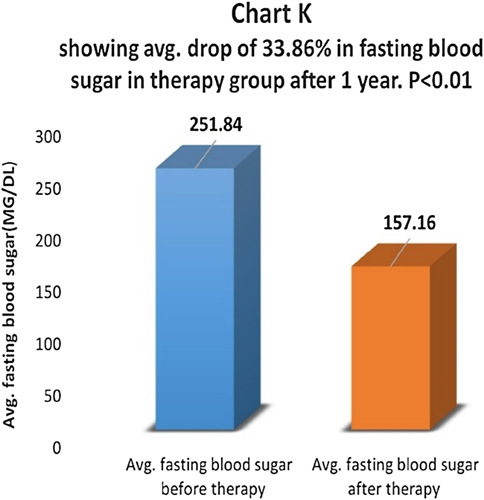


**Figure:**
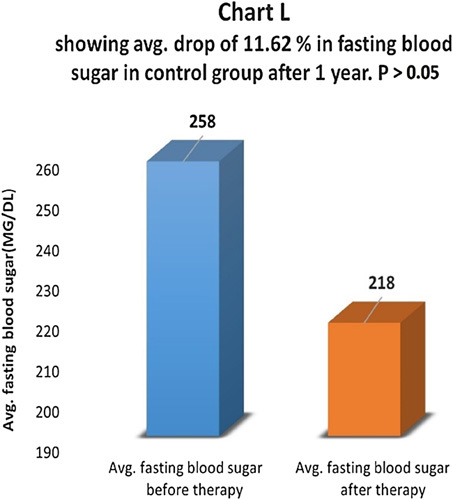


Postprandial blood sugar levels were compared before and one year after therapy in the therapy group (Chart M). There was an average drop of 39.46% in fasting blood sugar in the therapy group and the difference was statistically highly significant with *P*<0.01 (Chart N). In the control group, there was an average drop of 8.5% in postprandial blood sugar levels and the difference was not statistically significant, *P*>0.05 (Chart O). In the control group, 55.3% of patients had the same postprandial blood sugar, and 44.7% of patients had a higher postprandial blood sugar level.

**Figure:**
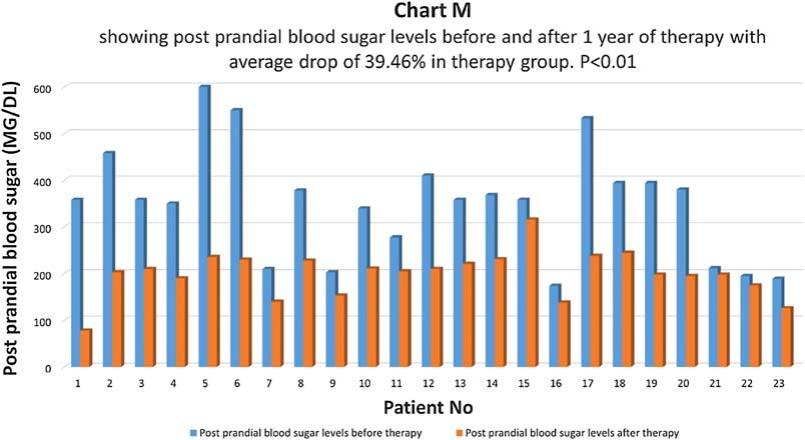


**Figure:**
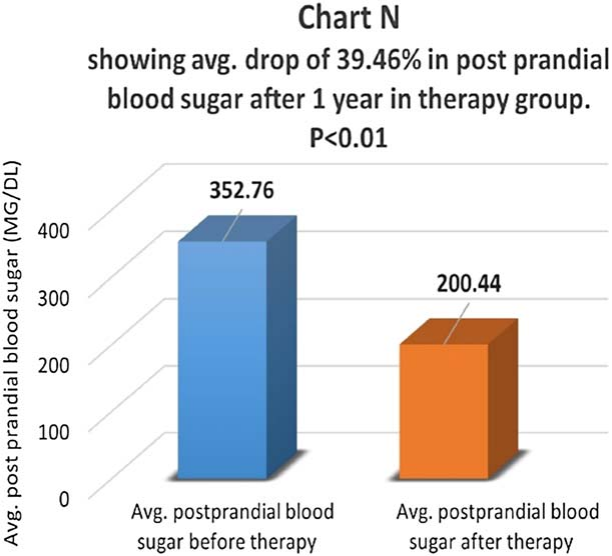


**Figure:**
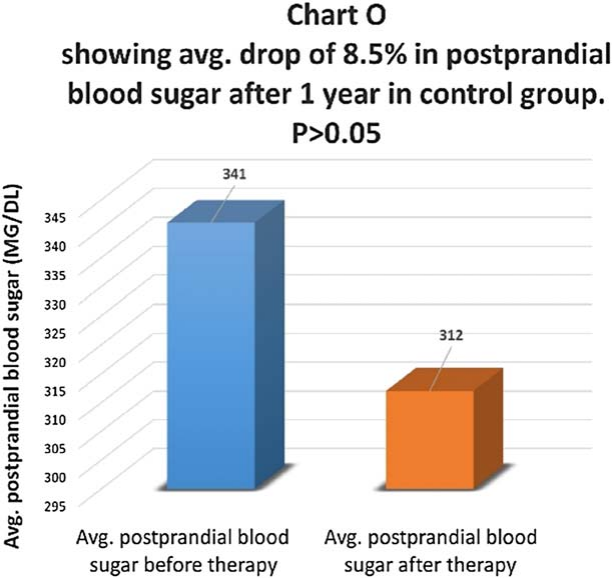


C-peptide levels were compared before and one year after therapy in the therapy group (Chart P). There was an average increase of 287.23% in C-peptide levels in the therapy group and the difference was statistically highly significant with *P*<0.01 (Chart Q). In the control group, there was an average increase of 3.19% in C-peptide levels and the difference was not statistically significant, *P*>0.05 (Chart R). In the control group, 75.1% of patients had the same C-peptide levels, and 24.9% of patients had a higher C-peptide level after therapy.

**Figure:**
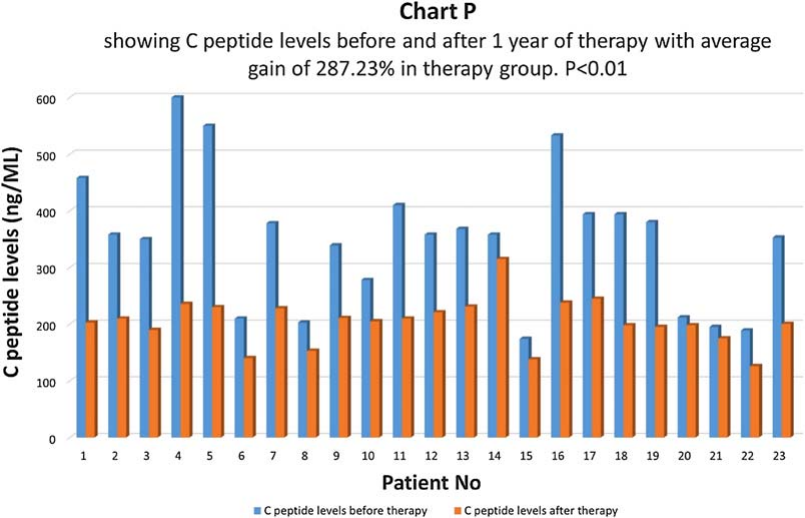


**Figure:**
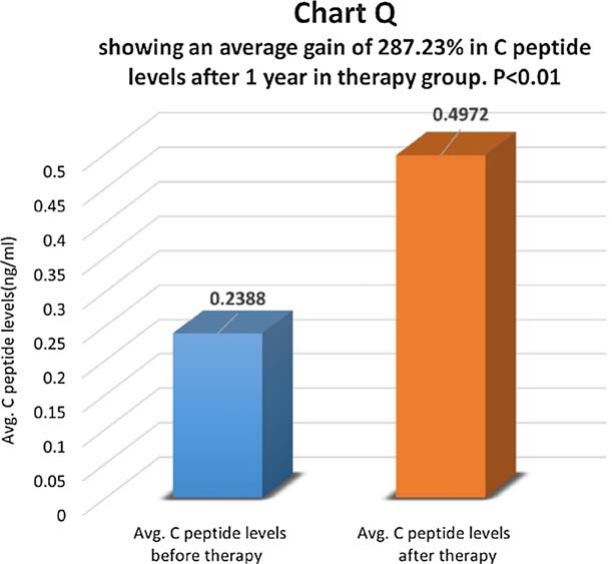


**Figure:**
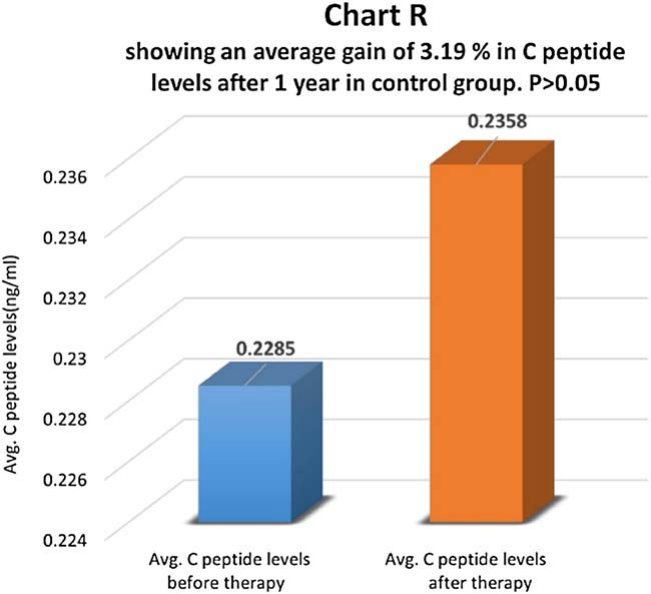


In the therapy group, anti-GAD antibody titer was measured before and after one year of therapy (Chart S). Only 6 patients (24%) had a higher-than-normal anti-GAD antibody titer which dropped to about 49.79% in one year, indicating a partial reversal of autoimmunity (Chart T). Since the number 6 is not adequate for statistical calculations, its significance was not calculated. In the control group, eight (30.76%) patients had a higher-than-normal anti-GAD antibody titer. At the end of one year, in the control group, anti-GAD antibody titer dropped by 6.07% (Chart U).

**Figure:**
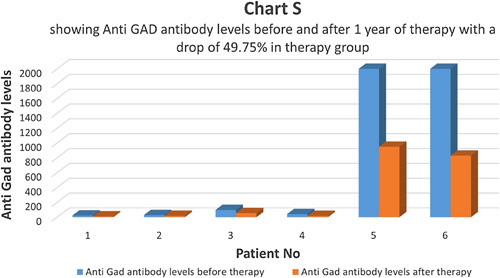


**Figure:**
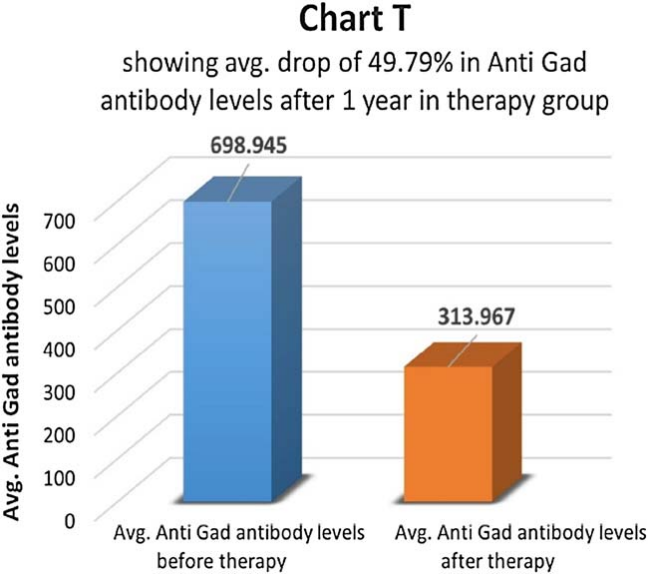


**Figure:**
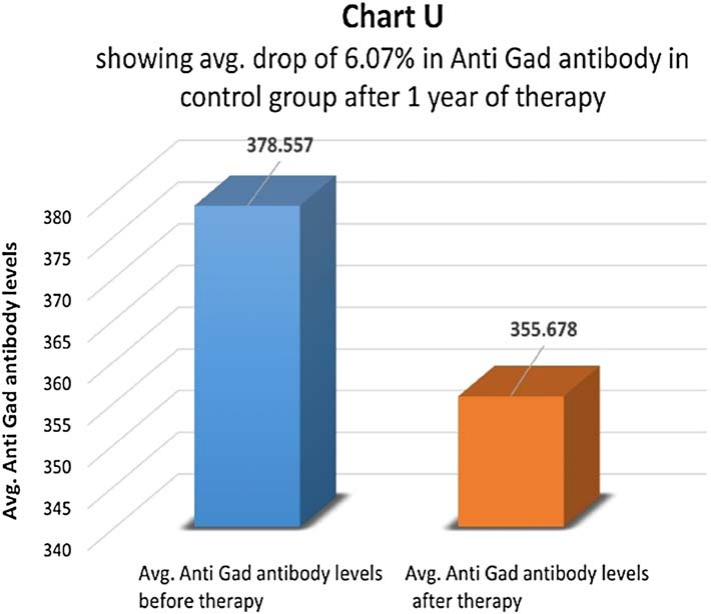


### Complications

In the above series, only minor complications and adverse reactions were noted, which were largely self-limiting and they are as follows (Table 1). Four patients (16%) had a mild skin rash that disappeared without any treatment. Five patients (20%) had pain in the abdomen and another 5 patients (20%) had postoperative pain, which completely resolved with a single dose of diclofenac Na injection as an analgesic. Two patients (8%) had mild nausea which did not require any treatment. Three (12%) patients had 2–5 cm inguinal ecchymosis, which was treated satisfactorily with Thrombophobe ointment application for 7 days. Only one (4%) patient had an inguinal hematoma which resolved with a Serrapeptase enzyme tablet given for 15 days postoperatively.

## Discussion

In the medical literature, many types of stem cells are described. These stem cells normally only replace one particular tissue; hence, they are called tissue-specific stem cells. They are also called adult stem cells. There are two types of adult stem cells^23^ mesenchymal and hemopoietic stem cells. The doubling time of stem cells is only 15.8 h^24^. They have the potential to duplicate indefinitely and differentiate into 22 types of cells^25^. Stem cells usually divide for up to 6 months inside the body and in the end, they turn into billions of cells. In case of organ damage, stem cells around the damaged area come forward and get divided and differentiated into specialized cells; thus, the damage is repaired. But in case of extensive damage, this process is inadequate and the organ starts failing. In stem cell therapy, we harvest stem cells from other healthy tissues as fat and bone marrow and put them into the diseased organ. These cells get divided and differentiated into specialized cells, and the organ starts functioning again. Stem cells from bone marrow cannot come into the various tissues due to the blood–bone marrow barrier. Similarly, stem cells from fat cannot migrate into diseased organs; hence, it is necessary to transplant them.

Stem cells produce many types of growth factors and cytokines^26^ which repair and differentiate adult tissues in an epigenetic manner. It means that these factors attach to the receptors on the cell surface and bring a positive change in the cell without entering the cell. That is called the paracrine function of stem cells for tissue repair. These growth factors and cytokines are released in vesicles called exosomes which release the factors to the target cells and regenerate them. Stem cells also self-renew themselves and others get differentiated into specialized cells when put in a particular organ due to growth factors and cytokines produced by that tissue. Mesenchymal stem cells (MSCs) have the ability to differentiate into tissues of all three lineages. This phenomenon is called plasticity^27–30^.

Type 1 diabetes is classified as an autoimmune disorder. But, in my series, there were only six patients (24%) with autoimmunity. There is one more hypothesis about the etiology of type 1 diabetes which states that the disease may be due to a one-time damage caused by a viral infection^31^. These patients without autoimmunity have no ongoing damage to the islets of Langerhans. Hence, they are likely to have a better and long-lasting results.

Autoimmune markers^32^ described in medical literature are autoantibodies to GAD, islet antigens (IA2 and IA2-beta), insulin, islet cells, and the zinc transporter ZnT8. The autoimmune marker used in this series was anti-GAD antibody titer since it was the only available test in my city. Serum C-peptide levels were used instead of insulin levels in the study for the following reasons. C-peptide and insulin are released from the pancreas in about equal amounts at the same time. Hence, a C-peptide test^33^ represents how much insulin is produced by your body. Since C-peptide tends to stay in the body longer than insulin, this test can be a good indirect way to measure insulin levels. Another fact in favor of C-peptide levels is that they are far more constant than insulin levels with far less fluctuations.

Studies have proved that by injecting stem cells intravenously, the autoimmunity can be reversed by the immunomodulatory function of MSCs^34,35^. In the above series, although stem cells are injected into the arterial supply of the pancreas, some of them escape from the venous side and the growth factors and cytokines released by them get attached to the T lymphocytes and can repair faulty signatures on them, thus helping to reverse autoimmunity. Studies^35^ have shown that adult MSCs suppress T-cell proliferation, cytokine secretion, and cytotoxicity and regulate the balance of Th1/Th2. They regulate the functions of regulatory T cells and increase B-cell viability but also may inhibit their proliferation and arrest the cell cycle. In addition, MSCs affect the secretion of antibodies and the production of co-stimulatory molecules of B cells. MSCs inhibit the maturation, activation, and antigen presentation of dendritic cells and also inhibit interleukin-2 (IL-2)-induced natural killer (NK) cell activation. Similar to adult MSCs also suppress the cytotoxic effects of activated NK cells and downregulate NK-activating receptors.

In a Petri dish, stem cells were grown in a hyperglycemic environment and after a few weeks they developed into islets-like cells which started producing insulin^36^. These islets were implanted in diabetic rats and they reversed diabetes and achieved euglycemia. When certain growth factors and cytokines were added into the culture of bone marrow-derived MSCs, these cells were transdifferentiated into islet-like cells which produced insulin *in vitro* ^36–39^. It is quite likely that the stem cells implanted in the pancreas can get differentiated into beta cells of the pancreas due to specific growth factors and cytokines such as fibroblast growth factor, epidermal growth factor, and vascular endothelial factor secreted by the pancreatic tissue.

There are published articles demonstrating the intrapancreatic administration of stem cells by cannulating the pancreatic artery by interventional radiology in type 2 diabetes patients^40,41^. But, in type 2 diabetes, the endogenous insulin production is either normal or higher than normal. It is the tissue resistance and the relative deficiency of insulin which are the root causes of type 2 diabetes. Hence, regenerating the existing islets of Langerhans and further increasing endogenous insulin production appears unnecessary to me. In type 1 diabetes, endogenous insulin production is greatly reduced. Hence, it makes great sense to repair and regenerate the islets of Langerhans in type 1 diabetes patients to increase endogenous insulin production.

There are many clinical trials that have reported good results of intravenously given stem cells in type 1 diabetes patients^42–46^. A study published recently documents that stem cells were injected in an Omental pouch, peritoneum, and blood of type 1 diabetes patients^47^. But, when given intravenously, very few stem cells reach the pancreas. Since intrapancreatic stem cell implantation may be risky for children under 10 years of age, I prefer to treat them with the above technique. Animal studies have shown that when stem cells are given intravenously, a vast majority of them are entrapped in the lungs^48,49^. Studies have also shown that the lungs engulf about 75% of the stem cells put in blood in the first circulation itself. Since the pancreas gets only 2% of blood volume, it gets only a small share of 2% of the remaining 25% of stem cells which were put into the blood. The remaining small fraction of stem cells are distributed to the various organs as per their percentage volume of the cardiac output received^50^. Since the pancreas receives less than 2% of the total cardiac output, the total stem cells received by it are negligible. Studies in the past have shown that the clinical results of stem cell therapy are directly proportional to the number of stem cells implanted into a particular organ. Animal studies done on rats have shown that the concentration of stem cells achieved in the pancreas is far higher when stem cells are injected directly into the pancreas through a pancreatic artery or by direct injection^51^. Since direct injection will be invasive and may lead to pancreatitis in humans, hence, I have opted for a much safer option of intrapancreatic implantation through the pancreatic artery by interventional radiology. Such therapy is 30–50 times more powerful than putting cells into the blood. The MSCs have a size of 30 μm^52^, about 4 times larger than the RBC size of 7.5 μm. They also have an irregular surface, unlike the smooth surface of RBCs. Hence, these stem cells are brought by arterioles into the tissue spaces where they are entrapped and they cannot escape from the venules out into the venous circulation. As we know, healthy RBCs pass out smoothly through the tissues. But when their shape gets abnormal, as in sickle cell anemia, they are trapped in the tissue spaces. The above argument is the foundation of intrapancreatic stem cell therapy for type 1 diabetes.

**Figure 3:**
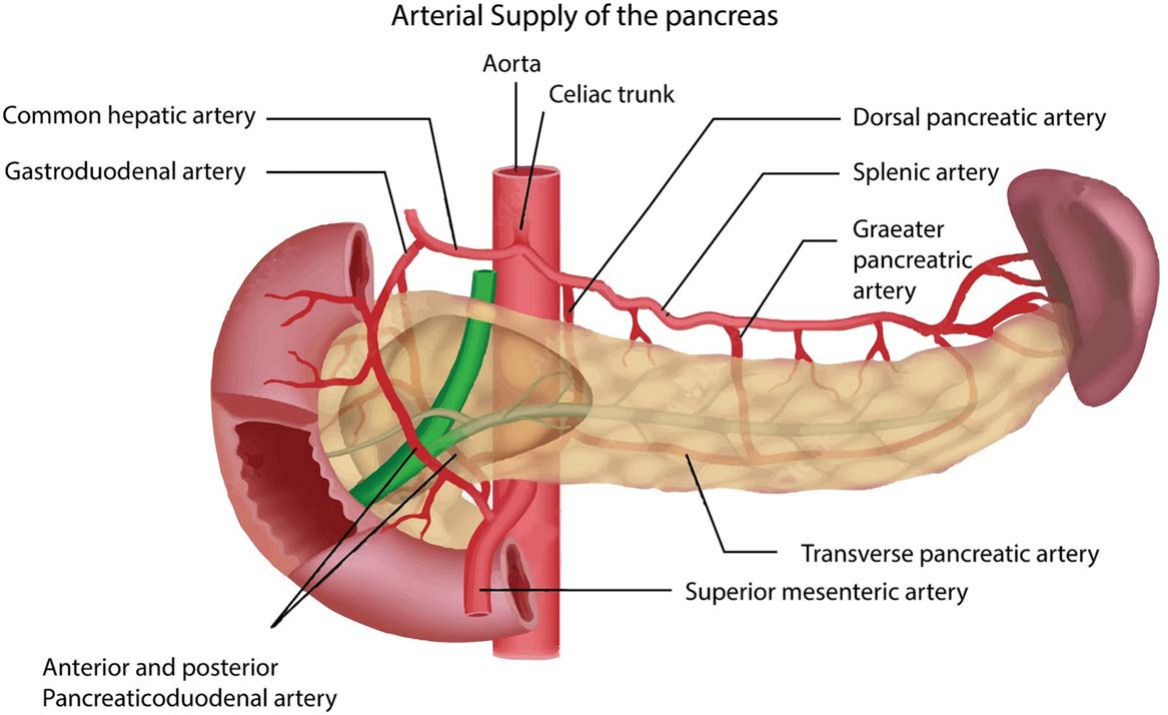
Blood supply of pancreas.

The pancreas has a dual blood supply^53^ (Fig. 3). It is supplied by the branches of the splenic artery (a branch of the celiac trunk), superior mesenteric artery (SMA), and the common hepatic artery. The gastroduodenal artery is a branch of the common hepatic artery. It supplies the head and the uncinate process of the pancreas in the form of the pancreaticoduodenal artery (PDA). The inferior PDA, which arises from the SMA, supplies a part of the inferior portion of the head of the pancreas. The body and the tail are supplied by the splenic artery and its branches, such as the dorsal pancreatic artery and transverse pancreatic artery. The pancreatic head drains into the superior mesenteric vein (SMV) and the body and the neck are drained by the splenic vein. The portal vein is formed by the merger of SMV and splenic vein.

The intrapancreatic stem cell implantation through the pancreatic artery was very challenging. Since the pancreas has a dual blood supply, half the quantity of isolated stem cells was injected into the main pancreatic artery and the remaining half was injected in the distal part of the splenic artery. Studies tell us that the majority (80%) of islets of Langerhans are located in the tail part of the pancreas^54^. Although the islets of Langerhans account for only 2% of the total volume of the pancreas, they are highly vascular and get 10–15% of the total blood supply of the pancreas^55^. Hence, half the quantity of the harvested stem cells was injected into the splenic artery near the pancreatic tail area. Patient no. 5,23 had spasms of the pancreaticoduodenal arteries making them invisible on angiography. Through the arterial catheter, nitroglycerine was injected in the arteries, and spasms disappeared after 10 min. In our series following anomalies of blood supply to the pancreas^19^ were noted. In patient no. 13,22,23, the celiac trunk had a stiff angle. Hence gastroduodenal artery could not be cannulated. Hence, stem cells were injected into the celiac artery itself.

The therapy group had substantially better results compared to the control group (Table 1). The variables to be compared in both groups were blood sugar fasting and postprandial, C-peptide levels, Anti-GAD antibody titer, Glycosylated Hb, and patient’s weight, along with total daily insulin requirement before and after therapy. The difference between the above variables was calculated before and after one year of the therapy and it became statistically highly significant. Autoimmunity was partially reversed in the therapy group. In children without autoimmunity, results were much better than in patients with autoimmunity. Patients with autoimmunity did not have good results. In the above series, only minor complications and adverse reactions were noted, which were largely self-limiting. Only three (12%) patients repeated the therapy after one year. They had a further 25–30% improvement in the variables compared. We need a study with a bigger number of patients repeating the therapy and its results because number 3 is a very small sample size and not statistically significant. By repeating the therapy multiple times with a gap of one year, we can hope that more patients can achieve euglycemia and insulin independence. The total treatment cost in my center was just USD 1000 instead of USD 50 000 in the USA. In some centers in India, stem cells are infused into the blood at the cost of USD 5000. That makes this therapy the cheapest stem cell therapy in the whole world.

The therapy has certain limitations, which are as follows. Type 1 diabetes patients have more than 80% damage to the beta cells of the pancreas at the time they are clinically diagnosed. By the time these patients come for this therapy, they have further damage with a little number of insulin-producing cells left. Many of these patients have very low C-peptide levels indicating practically negligible insulin production endogenously. Hence, although the growth factors and cytokines released by the implanted stem cells repair and regenerate the insulin-producing cells, only a little repair and regeneration is possible. This therapy can reverse autoimmunity only partially. Hence, the regenerated insulin-producing cells are vulnerable to ongoing damage due to autoimmunity.

But recently, many researchers have been able to take the same patients' autologous stem cells or unrelated donor’s stem cells, grow them in a laboratory *in vitro* and induce them into a huge number of insulin-producing beta cells with the help of certain transcription factors^56^. These cells can be implanted inside the pancreas or liver with the interventional radiology technique described in this article. This strategy can be a solution to the above limitation. Alternatively, these insulin-producing cells can be implanted into an Omental pouch described^47^. Since there is very little cellular immunity on the Omental surface compared to the blood, these cells can survive far longer, solving another limitation due to autoimmunity.

## Conclusions

Intrapancreatic stem cell therapy for type 1 diabetes is affordable, safe, and effective when compared to the control group with conventional treatment of type 1 diabetes. The results in the treatment group were statistically highly significant compared to the control group. The intrapancreatic infusion of stem cells was more effective than the intravenous group. The therapy shows great promise as a potential treatment for type 1 diabetes. Since the therapy can be repeated a number of times, it creates a lot of hope for type 1 diabetes patients. My stem cell therapy research is successful at a district place instead of a tertiary health care center. Hence, doctors in India and worldwide can replicate my success in small district-level centers, creating a lot of hope for millions of patients. We need further research on this therapy with a larger sample size and a longer follow-up time to determine the effectiveness of this management strategy and its possible complications.

## Ethical approval

Ethical committee approval was taken before starting the study.

## Consent

A well-written and informed consent was taken from all patients and their relatives.

## Sources of funding

This research did not receive any specific grant from funding agencies in the public, commercial, or not-for-profit sectors.

## Author contribution

Single author, no co-authors.

## Conflicts of interest disclosure

The authors declare that they have no conflicts of interest.

## Provenance and peer review

Not commissioned, externally peer-reviewed.

## References

[1] Mobasseri M Shirmohammadi M Amiri T . Prevalence and incidence of type 1 diabetes in the world: a systematic review and meta-analysis. Health Promot Perspect 2020;10:98–115. 32296622 10.34172/hpp.2020.18 PMC7146037

[2] Kumar KM . Incidence trends for childhood type 1 diabetes in India. Indian J Endocrinol Metab 2015;19(suppl 1):S34–S35. 25941646 10.4103/2230-8210.155378 PMC4413385

[3] Matsumoto S . Islet cell transplantation for type 1 diabetes. J Diabetes 2010;2:16–22. 20923470 10.1111/j.1753-0407.2009.00048.x

[4] Carlson RV Boyd KM Webb DJ . The revision of the Declaration of Helsinki: past, present and future. Br J Clin Pharmacol 2004;57:695–713. 15151515 10.1111/j.1365-2125.2004.02103.x PMC1884510

[5] American Diabetes Association. Diagnosis and classification of diabetes mellitus. Diabetes Care 2010;33(suppl 1):S62–S69. 20042775 10.2337/dc10-S062 PMC2797383

[6] Kawasaki E . Type 1 diabetes and autoimmunity. Clin Pediatric Endocrinol 2014;23:99–105. 10.1297/cpe.23.99 PMC4219937 25374439

[7] Lahiry S Choudhury S Sinha R . The National Guidelines for Stem Cell Research (2017): What academicians need to know? Perspect Clin Res 2019;10:148–154. 31649863 10.4103/picr.PICR_23_18 PMC6801994

[8] Indian Council of Medical Research. Guidelines for Stem Cell Research and Therapy 2007. Indian Council of Medical Research, 2007 [Accessed 28 January 2018]. http:// www.icmr.nic.in/stem_cell/stem_cell_guidelines_2007.pdf

[9] Jawale S Bhaskar V Nandikolmath V . Autologous stem cell therapy for cerebral palsy. Open J Pediatr 2020;10:36–64.

[10] Di Nicola V . Omentum a powerful biological source in regenerative surgery. Regen Ther 2019;11:182–191. 31453273 10.1016/j.reth.2019.07.008 PMC6700267

[11] Shah S Lowery E Braun RK . Cellular basis of tissue regeneration by omentum. PLoS One 2012;7:e38368. 22701632 10.1371/journal.pone.0038368 PMC3368844

[12] Hao T Chen J Zhi S . Comparison of bone marrow-vs. adipose tissue-derived mesenchymal stem cells for attenuating liver fibrosis. Exp Ther Med 2017;14:5956–5964. 29285145 10.3892/etm.2017.5333 PMC5740792

[13] Bunnell BA Flaat M Gagliardi C . Adipose-derived stem cells: isolation, expansion and differentiation. Methods 2008;45:115–120. 18593609 10.1016/j.ymeth.2008.03.006 PMC3668445

[14] Gorin NC Carreras E Dufour C . Bone marrow harvesting for HSCT The EBMT Handbook: Hematopoietic Stem Cell Transplantation and Cellular Therapies, 7th ed. Springer; 2019. 32091673

[15] Pierini M Dozza B Lucarelli E . Efficient isolation and enrichment of mesenchymal stem cells from bone marrow. Cytotherapy 2012;14:686–693. 22574721 10.3109/14653249.2012.677821

[16] Baglioni S Francalanci M Squecco R . Characterization of human adult stem-cell populations isolated from visceral and subcutaneous adipose tissue. FASEB J 2009;23:3494–3505. 19584303 10.1096/fj.08-126946

[17] Cornell RF Hari P Drobyski WR . Engraftment syndrome following autologous stem cell transplantation – an update unifying the definition and management approach. Biol Blood Marrow Transplant 2015;21:2061–2068. 26327628 10.1016/j.bbmt.2015.08.030 PMC4639405

[18] Holland BH Applegate RJ . Femoral Vascular Access – Approaches and Available Devices. Interventional Cardiology: Reviews, Research, Resources (ICR^3^).

[19] Witte B Fröber R Linss W . Unusual blood supply to the pancreas by a dorsal pancreatic artery. Surg Radiol Anat 2001;23:197–200. 11490932 10.1007/s00276-001-0197-5

[20] Bain BJ . Bone marrow biopsy morbidity: review of 2003. J Clin Pathol 2005;58:406–408. 15790706 10.1136/jcp.2004.022178 PMC1770618

[21] Waszczuk-Gajda A Penack O Sbianchi G . Complications of autologous stem cell transplantation in multiple myeloma: results from the CALM study. J Clin Med 2022;11:3541. 35743620 10.3390/jcm11123541 PMC9225651

[22] Tenny S Abdelgawad I . Statistical Significance. Stat Pearls Publishing; 2022. 29083828

[23] Nature Reports Stem Cells (2007) Published: 14 June 2007. What are the major types of stem cells?.

[24] Zhan X-S El-Ashram S Luo D-Z . A comparative study of biological characteristics and transcriptome profiles of mesenchymal stem cells. Int J Mol Sci 2019;20:1485. 30934541 10.3390/ijms20061485 PMC6471769

[25] Biehl JK Russell B . Introduction to stem cell therapy. J Cardiovasc Nurs 2009;24:98–105. 19242274 10.1097/JCN.0b013e318197a6a5 PMC4104807

[26] Majka M Janowska-Wieczorek A Ratajczak J . Numerous growth factors, cytokines, and chemokines are secreted by human CD34(+) cells, myeloblasts, erythroblasts, and megakaryoblasts and regulate normal hematopoiesis in an autocrine/paracrine manner. Blood 2001;97:3075–3085. 11342433 10.1182/blood.v97.10.3075

[27] Baraniak PR McDevitt TC . Stem cell paracrine actions and tissue regeneration. Regen Med Regen Med 2010;5:121–143. 20017699 10.2217/rme.09.74 PMC2833273

[28] Zakrzewski W Dobrzyński M Szymonowicz M . Stem cells: past, present, and future. Stem Cell Res Ther 2019;10:68. 30808416 10.1186/s13287-019-1165-5 PMC6390367

[29] Grove JE Bruscia E Krause DS . Plasticity of bone marrow-derived stem cells. Stem Cells 2004;22:487–500. 15277695 10.1634/stemcells.22-4-487

[30] Lakshmipathy U Verfaillie C . Stem cell plasticity. Blood Rev 2005;19:29–38. 15572215 10.1016/j.blre.2004.03.001

[31] Coppieters KT Boettler T von Herrath M . Virus infections in type 1 diabetes. Cold Spring Harb Perspect Med 2012;2:a007682. 22315719 10.1101/cshperspect.a007682 PMC3253029

[32] Taplin CE Barker JM . Autoantibodies in type 1 diabetes. Autoimmunity 2008;41:11–18. 18176860 10.1080/08916930701619169

[33] Leighton E Sainsbury CA Jones GC . A practical review of c-peptide testing in diabetes. Diabetes Ther 2017;8:475–487. 28484968 10.1007/s13300-017-0265-4 PMC5446389

[34] Gao F Chiu SM Motan DAL . Mesenchymal stem cells and immunomodulation: current status and future prospects. Cell Death Dis 2016;7:e2062. 26794657 10.1038/cddis.2015.327 PMC4816164

[35] Weiss ARR Dahlke MH . Immunomodulation by mesenchymal stem cells (MSCs): mechanisms of action of living, apoptotic, and dead MSCs. Front Immunol 2019;10:1191. 31214172 10.3389/fimmu.2019.01191 PMC6557979

[36] Oh S-H Muzzonigro TM Bae S-H . Adult bone marrow-derived cells trans-differentiating into insulin-producing cells for the treatment of type I diabetes. Lab Invest 2004;84:607–17. 15034596 10.1038/labinvest.3700074

[37] Luo LG Luo JZQ Xiong F . Cytokines inducing bone marrow SCA+ cells migration into pancreatic islet and conversion into insulin-positive cells in vivo. PLoS One 2009;4:e4504. 19225560 10.1371/journal.pone.0004504 PMC2637986

[38] Oh S-H Muzzonigro TM Bae S-H . Adult bone marrow-derived cells trans-differentiating into insulin-producing cells for the treatment of type I diabetes. Lab Invest 2004;84:607–617. 15034596 10.1038/labinvest.3700074

[39] Khatri R Mazurek S Petry SF . Mesenchymal stem cells promote pancreatic β-cell regeneration through downregulation of FoxO1 pathway. Stem Cell Res Ther 2020;11:497. 33239104 10.1186/s13287-020-02007-9 PMC7687794

[40] Estrada EJ Valacchi F Nicora E . Combined treatment of intrapancreatic autologous bone marrow stem cells and hyperbaric oxygen in type 2 diabetes mellitus. Cell Transplant 2008;17:1295–1304. 19364067 10.3727/096368908787648119

[41] Gao S Zhang Y Liang K . Mesenchymal stem cells (MSCs): a novel therapy for type 2 diabetes. Stem cell Int 2022;2022:8637493. 10.1155/2022/8637493 PMC9424025 36045953

[42] Bhartiya D . Stem cells to replace or regenerate the diabetic pancreas: huge potential & existing hurdles. Indian J Med Res 2016;143:267–274. 27241638 10.4103/0971-5916.182615 PMC4892071

[43] Armand P Fedik. AR Wibisono S . Autologous MSC bone marrow stem cell and allogenic pancreatic stem cell for repair of beta pancreatic cell in experimental diabetes mellitus. Afr J Intern Med 2012;1:10–16.

[44] Sabry D Marzouk S Zakaria R . The effect of exosomes derived from mesenchymal stem cells in the treatment of induced type 1 diabetes mellitus in rats. Biotechnol Lett 2020;42:1597–1610. 32430801 10.1007/s10529-020-02908-y

[45] Phuc Pham Van. Stem Cell Therapy for Islet Regeneration. Submitted: October 21st 2010. Reviewed: April 4th 2011. Published: August 23rd 2011. doi: 10.5772/17588

[46] Ho JH Tseng T-C Ma W-H . Multiple intravenous transplantations of mesenchymal stem cells effectively restore long-term blood glucose homeostasis by hepatic engraftment and β-cell differentiation in streptozocin-induced diabetic mice. Cell Transplant 2012;21:997–1009. 22004871 10.3727/096368911X603611

[47] Jawale S . Stem cell therapy for type1 diabetes with transplantation of stem cells into the Omental pouch, peritoneum, and blood, experimental study. Ann Med Surg (Lond) 2022;81:104468. 36147094 10.1016/j.amsu.2022.104468 PMC9486716

[48] Fischer. UM Harting MT Jimenez F . Pulmonary passage is a major obstacle for intravenous stem cell delivery: the pulmonary first-pass effect. Stem Cells Dev 2009;18:683–691. 19099374 10.1089/scd.2008.0253 PMC3190292

[49] Schrepfer S Deuse T Reichenspurner H . Stem cell transplantation: the lung barrier. Transplant Proc 2007;39:573–576. 17362785 10.1016/j.transproceed.2006.12.019

[50] Gao J Dennis JE Muzic RF . The dynamic in vivo distribution of bone marrow-derived mesenchymal stem cells after infusion. Cells Tissues Organs 2001;169:12–20. 11340257 10.1159/000047856

[51] Khatri R Petry SF Linn T . Intrapancreatic MSC transplantation facilitates pancreatic islet regeneration. Stem Cell Res Ther 2021;12:121. 33579357 10.1186/s13287-021-02173-4 PMC7881671

[52] Liu L Tseng L Ye Q . A new method for preparing mesenchymal stem cells and labeling with ferumoxytol for cell tracking by MRI. Sci Rep 2016;6:26271. 27188664 10.1038/srep26271 PMC4870722

[53] Kulenović A Sarač-Hadžihalilović A . Blood vessels distribution in body and tail of pancreas - a comparative study of age-related variation. Bosn J Basic Med Sci 2010;10:89–93. 10.17305/bjbms.2010.2700 PMC5509407 20507286

[54] Wang X Misawa R Zielinski MC . Regional Differences in Islet Distribution in the Human Pancreas - Preferential Beta-Cell Loss in the Head Region in Patients with Type 2 Diabetes. PLoS One 2013;8:e67454. 23826303 10.1371/journal.pone.0067454 PMC3691162

[55] Islets of Langerhans - an overview | ScienceDirect Topics. https://www.sciencedirect.com/topics/neuroscience/islets-of-langerhans

[56] Silva IBB Kimura CH Colantoni VP . Stem cells differentiation into insulin-producing cells (IPCs): recent advances and current challenges. Stem Cell Res Ther 2022;13:309. 35840987 10.1186/s13287-022-02977-y PMC9284809

